# High-quality draft genome sequence of *Effusibacillus lacus* strain skLN1^T^, facultative anaerobic spore-former isolated from freshwater lake sediment

**DOI:** 10.1186/s40793-017-0302-y

**Published:** 2017-12-13

**Authors:** Miho Watanabe, Riho Tokizawa, Hisaya Kojima, Manabu Fukui

**Affiliations:** 1 Institute of Low Temperature Science, Hokkaido University, Nishi 8, Kita 19, Kita-ku, Sapporo, Hokkaido 060-0819 Japan; 2 Postdoctoral Research Fellow of the Japan Society for the Promotion of Science, Chiyoda-ku, Tokyo, 102-8471 Japan

**Keywords:** Draft genome sequence, Spore forming bacteria, The family *Alicyclobacillaceae*, The genus *Effusibacillus*

## Abstract

10.1601/nm.25721 strain skLN1^T^ is the type strain of the type species in the genus 10.1601/nm.25720 which is the one of the genera in the family 10.1601/nm.5070 within the phylum 10.1601/nm.3874. 10.1601/nm.25721 strain skLN1^T^ is a Gram-positive, spore-forming thermophilic neutrophile isolated from freshwater lake sediment. Here, we present the draft genome sequence of strain skLN1^T^, which consists of 3,902,380 bp with a G + C content of 50.38%.

## Background

The family *Alicyclobacillaceae* consists of four genera; *Alicyclobacillus*, *Kyrpidia* *,* *Tumebacillus* and Effusibacillus. *Alicyclobacillus* spp. are known as the significant causative microorganisms of fruit juice spoilage [1, 2] *Kyrpidia tusciae*, a sole characterized species of the genus *Kyrpidia* is a thermoacidophile which grows best under autotrophic conditions [3, 4]. Members of the genus *Tumebacillus* are mesoneutrophile which are derived from various environments, such as the Arctic permafrost, wastewater and and soil [5–7]. Genus 10.1601/nm.25720 was established in this family together with the reclassification of *Alicyclobacillus pohliae* as *Effusibacillus pohliae* and *Alicyclobacillus consociatus* as *Effusibacillus consociatus* [8]. *Effusibacillus lacus* strain skLN1^T^ is a facultative anaerobic, Gram-positive bacterium isolated from freshwater lake sediment. Here, we descibe draft genome sequence of the type strain of this genus, *Effusibacillus lacus* strain skLN1^T^. In this study, we summarize the features of *E. lacus* strain skLN1^T^ and show an overview of draft genome sequence and annotation of this strain.

## Organism information

### Classification and features

**Fig. 1:**
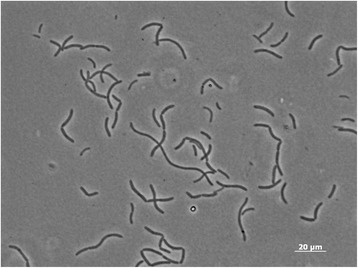
Photomicrograph of cells of *Effusibacillus lacus* strain skLN1^T^. Cells were grown on aerobic R2A liquid medium at 50 °C for 1 day

*E. lacus* strain skLN1^T^ was isolated from sediments of a freshwater lake, Lake Yamanashi, Japan [8]. Cell wall structure of this strain is Gram-positive type. Cells of this strain are spore-forming rods varied from 5 to 100 μm in length (Fig. 1, Table 1). The major cellualr fatty acids of this strain are iso-C _14 : 0_, iso-C _15 : 0_ and iso-C _16 : 0_. Respiratory quinones of this strain are MK-7 (99.5%) and MK-8 (0.5%). The cell-wall peptidoglycan of this strain consists of meso-diaminopimelic acid, alanine and glutamic acid, indicating the presence of A1γ-type polymer. This bacterium is facultative anaerobe and is capable of respiration and fermentation. Sugars, organic acids, peptides and amino acids are used for fermentative growth of this strain. Strain skLN1^T^ reduce nitrate to nitrite under anaerobic conditions in the presence of lactate. This strain cannot grow lithoautotrophically with elemental sulfur or thiosulfate under oxic/anoxic conditions in the presence nitrate.

**Table 1:** Classification and general features of *Effusibacillus lacus* strain skLN1^T^ according to MIGS recommendations

MIGS ID	Property	Term	Evidence code^a^
	Classification	Domain *Bacteria*	TAS [9]
		Phylum *Firmicutes*	TAS [18, 19]
		Class *Bacilli*	TAS [20]
		Order *Bacillaceae*	TAS [21, 22]
		Family *Alicyclobacillales*	TAS [3, 23]
		Genus *Effusibacillus*	TAS [8]
		Species *Effusibacillus lacus*	TAS [8]
		Type strain: skLN1^T^ (BDUF00000000)	
	Gram stain	Variable	TAS [8]
	Cell shape	Rod	TAS [8]
	Motility	Motile	TAS [8]
	Sporulation	Spore-forming	TAS [8]
	Temperature range	28–60 °C	TAS [8]
	Optimum temperature	50–52 °C	TAS [8]
	pH range; Optimum	7.0–8.5; 7.25–7.5	TAS [8]
	Carbon source	Organic acids, sugars, peptones, amino acids	TAS [8]
MIGS-6	Habitat	freshwater lake sediment	TAS [8]
MIGS-6.3	Salinity	0% NaCl (*w*/*v*)	TAS [8]
MIGS-22	Oxygen requirement	Facultatively anaerobic	TAS [8]
MIGS-15	Biotic relationship	Free-living	NAS
MIGS-14	Pathogenicity	None	NAS
MIGS-4	Geographic location	Yamanashi, Japan	TAS [8]
MIGS-5	Sample collection	March 2009	NAS
MIGS-4.1 MIGS-4.2	Latitude-Longitude	not reported	NAS
MIGS-4.4	Altitude	not reported	NAS

^a^Evidence codes - TAS Traceable Author Statement (i.e., a direct report exists in the literature), NAS Non-traceable Author Statement (i.e., not directly observed for the living, isolated sample, but based on a generally accepted property for the species, or anecdotal evidence). *NA* not avairable

**Fig. 2:**
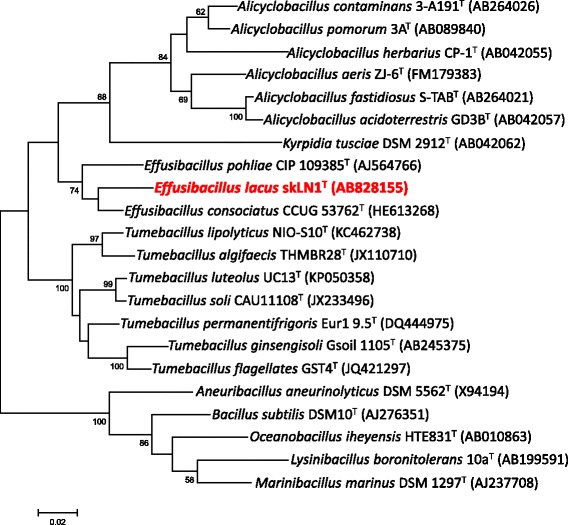
Phylogenetic tree showing the relationship of *Effusibacillus lacus* strain skLN1^T^ and related representatives. The maximum-likelihood tree was constructed with MEGA version 7.0.20 [24] based on ClustalX version 2.1 [25] aligned sequences of 16S rRNA gene. Bootstrap values (percentages of 1000 replications) of ≥50% are shown at nodes

The phylogenetic position of *E. lacus* strain skLN1^T^ among the members of the family *Alicyclobacillaceae* is shown in the phylogenetic tree based on the 16S rRNA gene sequence (Fig. 2). Strain skLN1^T^, *E. consociatus* and *E. pohliae* are classified into an independent cluster in the family *Alicyclobacillaceae* *.*

## Genome sequencing information

### Genome project history

*E. lacus* strain skLN1^T^ was selected for genome sequencing on the basis of its 16S rRNA gene-based phylogenetic position in the family *Alicyclobacillaceae* (Fig. 2). Table 2 shows a summary of the genome sequencing project information and its association with MIGS version 2.0 compliance [9]. The genome consists of 127 contigs, which has been deposited at DDBJ/EMBL/GenBank under accession number BDUF01000000.

**Table 2:** Project information

MIGS ID	Property	Term
MIGS 31	Finishing quality	High-quality draft
MIGS-28	Libraries used	TruSeq Nano DNA library prep kit
MIGS 29	Sequencing platforms	Illumina Hiseq paired-end
MIGS 31.2	Fold coverage	282×
MIGS-30	Assemblers	Velvet version 1.2.08
MIGS 32	Gene calling method	MetaGene
	Locus Tag	EFBL
	Genbank ID	BDUF00000000
	Genbank Date of Release	September 13, 2017
	GOLD ID	NA
	BIOPROJECT	PRJDB5819
MIGS 13	Source Material Identifier	SAMD00081395, DSM 27172
	Project relevance	Ecology and evolution

### Growth conditions and genomic DNA preparation

10.1601/nm.25721 strain skLN1^T^ (10.1601/strainfinder?urlappend=%3Fid%3DDSM+27172) was grown aerobically on TSB liquid medium (Daigo) at 50 °C without shaking. Genomic DNA was extracted from collected cells using Wizard® genomic DNA purification kit (Promega).

### Genome sequencing and assembly

The genome sequence of strain skLN1^T^ was determined using paired-end Illumina sequencing at Hokkaido System Science Co., Ltd. (Japan). The 11,205,386 reads were generated from a library with 100 bp inserts. After trimming of the reads, a total of 11,009,340 high-quality filtered paired end reads with a hash length of 95 bp were obtained. Reads were assembled de novo using Velvet version 1.2.08 into 127 scaffolds.

### Genome annotation

vhThe genome sequence of strain skLN1^T^ was automatically annotated and analyzed through the MiGAP pipeline [10]. In this pipeline, RNAmmer [11] and tRNAscan-SE [12] were used to identify rRNA and tRNA genes, respectively. MetaGene Annotator [13] was used for prediction of open reading frames likely to encode proteins (coding sequences), and functional annotation was performed based on reference databases, including Reference Sequence, TrEMBL, and Clusters of Orthologous Groups. Manual annotation was performed using IMC-GE software (In Silico Biology; Yokohama, Japan). Putative CDSs possessing BLASTP matches with more than 70% coverage, 35% identity and E-values less than 1 × e^−5^ were considered potentially functional genes. The CDSs were annotated as hypothetical proteins when these standard values were not satisfied. Transcription start sites of predicted proteins were corrected based on multiple sequence alignments. The protein-coding genes in the genome were also subjected to analysis on WebMGA [14] for the COGs and Protein family annotations. Transmembrane helices and signal peptides were predicted by using Phobius [15]. CRISPR loci were distinguished using the CRISPR Recognition Tool [16]. General features of *Effusibacillus lacus* strain skLN1^T^ and the MIxS mandatory information were show in Table 1.

## Genome properties

The total genome of *E. lacus* strain skLN1^T^ was 3,902,380 bp in size with a GC content of 50.38% (Table 3). It was predicted to contain 3733 genes including 3683 protein-coding genes and 50 RNA genes (for tRNA). Approximately 77.5% of the predicted genes were assigned to COG functional categories. The distribution of genes into COGs functional categories is presented in Table 4.

**Table 3:** Genome statistics

Attribute	Value	% of Total
Genome size (bp)	3,902,380	100
DNA coding (bp)	3,237,729	82.97
DNA G + C (bp)	1,966,019	50.38
DNA scaffolds	127	–
Total genes	3733	100
Protein coding genes	3683	98.66
RNA genes	50	1.34
Pseudo genes	NA	NA
Genes in internal clusters	NA	NA
Genes with function prediction	2588	69.33
Genes assigned to COGs	2893	77.50
Genes with Pfam domains	3111	83.34
Genes with signal peptides	434	11.63
Genes with transmembrane helices	799	21.40
CRISPR repeats	2	–

*NA* not avairable

**Table 4:** Number of genes associated with general COG functional categories

Code	count	%age	description
J	165	4.42	Translation, ribosomal structure and biogenesis
A	0	0.00	RNA processing and modification
K	243	6.51	Transcription
L	146	3.91	Replication, recombination and repair
B	1	0.03	Chromatin structure and dynamics
D	42	1.13	Cell cycle control, cell division, chromosome partitioning
V	30	0.80	Defense mechanisms
T	194	5.20	Signal transduction mechanisms
M	178	4.77	Cell wall/membrane/envelope biogenesis
N	76	2.04	Cell motility
U	69	1.85	Intracellular trafficking, secretion, and vesicular transport
O	125	3.35	Posttranslational modification, protein turnover, chaperones
C	241	6.46	Energy production and conversion
G	176	4.71	Carbohydrate transport and metabolism
E	341	9.13	Amino acid transport and metabolism
F	74	1.98	Nucleotide transport and metabolism
H	165	4.42	Coenzyme transport and metabolism
I	153	4.10	Lipid transport and metabolism
P	177	4.74	Inorganic ion transport and metabolism
Q	83	2.22	Secondary metabolites biosynthesis, transport and catabolism
R	402	10.77	General function prediction only
S	271	7.26	Function unknown
–	840	22.50	Not in COGs

## Insights from the genome sequence

*E. lacus* strain skLN1^T^ possesses genes of key enzymes for dissimilatory nitrate reduction, i.e. *napA* (locus tag: EFBL_1421), *narGHJI* (EFBL_3070–3073), *nirK* (EFBL_0113), *norB* (EFBL_3053), *nrfA* (EFBL_2499) and related genes. Both genes for membrane-bound and periplasmic nitrate reductases (*narG* and *napA*) were identified in the genome. A protein coded in the 61,298–63,379 bp region of contig095 showed high amino-acid sequence similarity (≤ 74%) to nitrous-oxide reductase (Nos*Z*), although the region was not annotated as *nosZ* gene because of the internal assembly gaps. Genome of *E. lacus* strain skLN1^T^ contains the genes for complete denitrification to N_2_ gas (*nirK*, *norB* and *nosZ*) and dissimilatory ammonification (*nrfA*), although end product of nitrate reduction identified in the previous study was nitrite [8]. The reduction of nitrate to nitrite was reported in several species in the family *Alicylobacillaceae*, but denitrifying organisms have not been reported in this family. Genetic components involved in dissimilatory nitrate reduction were not found in the genome of *Effucibacillus pohliae* strain 10.1601/strainfinder?urlappend=%3Fid%3DDSM+22757 ^T^. *Kyrpidia tuscia* *e* 10.1601/strainfinder?urlappend=%3Fid%3DDSM+2912 ^T^ possesses *norB* gene, but genes for the other denitrification enzymes were not found in the genome of this strain [17]. Additionally, genes for dissimilatory sulfur oxidation were not identified in the genome of *E. lacus* strain skLN1^T^, although this organism was isolated from a sulfur-oxidizing enrichment culture [8].

## Conclusions

This study contributed to the knowledge of genome sequences of the genus 10.1601/nm.25720 within the family *Alicyclobacillaceae*. The genome of *E. lacus* strain skLN1^T^ consists of 3683 protein-coding genes and 50 RNA genes. Genes involved in dissimilatory nitrate reduction were identified in the genome of this organism.

## Abbreviations

CRISPR: Clustered regularly interspaced short palindromic repeat
MiGAP: Microbial genome annotation pipeline
nap: Periplasmic nitrate reductase
nar: Respiratory nitrate reductase
nir: Nitrite reductase
nor: Nitric oxide reductase
nos: Nitrous oxide reductase
nrf: Ammonia-forming cytochrome c nitrite reductase subunit c552
